# Recent Advances in Clinical Research for Skin Cancer Chemoprevention

**DOI:** 10.3390/cancers15153819

**Published:** 2023-07-27

**Authors:** Ruby Tow, Samuel Hanoun, Bradley Andresen, Ayaz Shahid, Jeffrey Wang, Kristen M. Kelly, Frank L. Meyskens, Ying Huang

**Affiliations:** 1 Department of Pharmaceutical Sciences, College of Pharmacy, Western University of Health Sciences, Pomona, CA 91766, USA; ruby.tow@westernu.edu (R.T.); samuel.hanoun@westernu.edu (S.H.); bandresen@westernu.edu (B.A.); ashahid@westernu.edu (A.S.); jwang@westernu.edu (J.W.); 2 Department of Dermatology, University of California, Irvine, CA 92697, USA; kmkelly@hs.uci.edu; 3 Departments of Medicine and Biological Chemistry, Chao Family Comprehensive Cancer Center, University of California, Irvine, CA 92868, USA; flmeyske@hs.uci.edu

**Keywords:** chemoprevention, skin cancer, NMSC, BCC, SCC, melanoma, UV, NSAID, nicotinamide

## Abstract

**Simple Summary:**

Skin cancer is the most common cancer type in the United States and the world. Both non-melanoma and melanoma skin cancer show a clear association with overexposure to solar ultraviolet radiation. Chemoprevention is an appealing strategy to control the increasing rate of skin cancer. Since the target population for cancer chemoprevention is healthy individuals with high cancer risk, pharmacological agents that can be used for preventive purposes should be both effective and safe. The present review outlines the current state of skin cancer chemoprevention clinical trials, in terms of study populations, agents, outcomes (including cancer risk reduction), predictive biomarkers, and adverse reactions. The most studied agents include non-steroidal anti-inflammatory drugs, retinoids, 5-fluorouracil, and nicotinamide. The route of administration can be oral or topical. Since the trial outcomes for most of these agents are inconsistent, there is a need for additional research in this area.

**Abstract:**

Neoplasm arising from the keratinocytes or melanocytes in the skin is the most prevalent type of cancer in the United States and worldwide. Since ultraviolet (UV) radiation may be a causing factor for several types of skin cancer, effective strategies to manage skin cancer include preventive measures such as minimizing exposure to UV and applying sunscreens. However, the effect of sunscreen in reducing skin cancer incidence remains uncertain. An alternative approach to prevent skin cancer is chemoprevention, which is defined as using either natural products or synthetic compounds to inhibit, delay, or reverse the development of cancer. Preclinical studies have demonstrated the effectiveness of multiple pharmacological agents and dietary supplements. However, whether preclinical findings can be translated into clinical application is unknown. This review evaluates the state of recent clinical trials investigating chemopreventive agents focusing on skin cancer to compare the target populations, interventions, endpoints, and outcomes of these trials. The ClinicalTrials and PubMed databases were searched for their available literature using the key words “skin cancer” and “chemoprevention”. The objective of this review is to provide updated information on the effectiveness and side effects of promising chemopreventive agents in human subjects and to identify research gaps.

## 1. Introduction

Skin cancer is one of the most common types of malignancy in the United States and the world, with rising incidence [1,2]. It is estimated that one out of five Americans will develop skin cancer by the age of 70, with Caucasians displaying the highest incidence [3]. Non-melanoma skin cancer (NMSC), including basal cell carcinoma (BCC) and squamous cell carcinoma (SCC), is the most common. Merkel cell carcinoma (MCC) less common, while malignant melanoma is one of the types of skin cancer with a higher potential for mortality. Skin cells can undergo neoplastic transformation due to DNA damage caused by environmental factors like exposure to ultraviolet (UV) radiation from sunlight or artificial sources like tanning beds and sunlamps [3]. UV not only induces DNA damage, but also creates an inflammatory and immunosuppressive microenvironment in which premalignant cells may grow into tumors [4].

**Figure 1:**
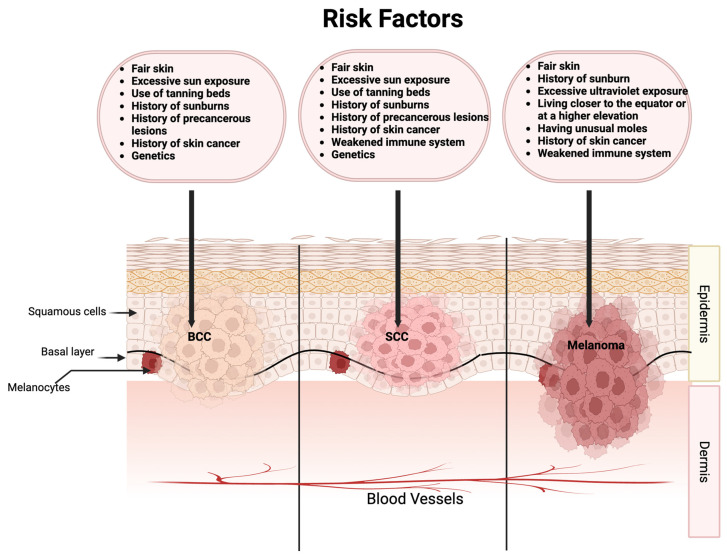
Risk factors for the most common types of skin cancer. BCC: basal cell carcinoma; SCC: squamous cell carcinoma.

BCC is the most common form of skin cancer, with an estimated 3.6 million cases yearly in the United States [5]. BCC is an uncontrolled and abnormal growth of skin cells in the epidermis, specifically the basal cell layer (Figure 1). BCCs are usually found on skin areas exposed to the sun, including the face, neck, ears, scalp, shoulders, and back. Besides UV radiation, risk factors for BCC include older age, male gender, fair skin, and outdoor careers [5]. Genetic mutations in the genes of the hedgehog pathway contribute to the majority of BCCs [5]. BCCs grow slowly, cause minimal damage, and rarely metastasize when detected and treated early. However, some lesions can be highly destructive if left untreated.

SCC is the second most prevalent type of skin cancer, with an estimated annual occurrence of 1.8 million cases within the United States [6]. SCC is an uncontrolled and abnormal growth of squamous cells that are in the epidermis layer (Figure 1). Like BCC, SCC is also found in similar places exposed to the sun, such as the face, ears, scalp, neck, and hands. SCCs share similar risk factors to BCC but with the addition of a weakened immune system, sun-sensitive conditions such as *Xeroderma pigmentosum*, skin precancers such as actinic keratosis (AK), and a history of human papillomavirus infection [7]. In particular, organ transplant recipients who receive long-term immunosuppressive treatment are at greater risk for skin cancer: for BCC, there is a 10-fold increased risk, while the risk of developing SCC is greater by 65–250 times [8]. Compared to BCC, SCC may grow relatively more rapidly and metastasize more quickly if not detected or treated early.

Melanoma or malignant melanoma develops from melanocytes, which are specialized cells responsible for producing pigment in the skin (Figure 1). Melanoma is a potentially lethal form of skin cancer. Over the past few decades, the incidence of melanoma is increasing in a rate more than any other malignancy in the United States [9]. Unlike other types of skin cancer, melanoma can grow in existing moles or may develop in various skin regions, even in areas not typically exposed to the sunlight. Risk factors for melanoma include UV exposure, weakened immune system, atypical moles, fair skin, skin cancer history, and family history of melanoma [10].

A rare type of skin cancer is MCC, which is an aggressive neuroendocrine carcinoma of the skin. The risk factors for MCC include older age, fair skin, extensive UV exposure, history of multiple skin cancers, and chronic immunosuppression due to HIV or solid organ transplantation [11]. About 80% of MCC is caused by Merkel cell polyomavirus infection, while 20% is caused by UV-mediated skin damage.

Other than immunosuppressants, recently, concerns have been raised about increased skin cancer risks associated with some commonly prescribed drugs: tumor necrosis factor alpha inhibitors, angiotensin-receptor blockers, phosphodiesterase type 5 inhibitors, and 3-hydroxy-3-methylglutaryl coenzyme A (HMG-CoA)-reductase inhibitors [12]. These drugs may interact with UV radiation, leading to photosensitivity responses in susceptible individuals [13].

In contrast to many other types of cancers, skin cancers emerge on the surface of the body, are usually visible, and thus, can be detected early by regular skin examinations at home and by dermatologists. Although the majority cases of NMSC can be cured with surgical excision, due to the high incidence, treatment of these diseases leads to a huge burden on health care systems [14]. Aside from early detection, managing the known risk factors contributing to skin cancer development is important, e.g., using UV-protective strategies such as avoiding excessive sun exposure and applying sunscreen. However, the implementation of UV-protective strategies is inconsistent; there is inadequate evidence as to whether sunscreen use can reduce the risk of skin cancer [14,15,16]. Cancer prevention has become one area of priority in “The War on Cancer” and represents a recently renewed goal aiming for reducing the cancer death rate by at least 50% over the next 25 years [17]. Chemoprevention, which is defined as the use of natural products or pharmacological agents to inhibit, block, or reverse cancer initiation, promotion, and progression, has also been investigated for skin cancer prevention [18,19]. The number of preclinical studies on skin cancer chemoprevention has been growing. However, there are few clinical studies able to provide sufficient evidence for recommending the use of chemopreventive agents for high-risk individuals.

This review aims to examine the status of recent skin cancer chemoprevention clinical trials regarding target populations, interventions, mechanisms of action, biomarkers, and outcomes. The ClinicalTrials and PubMed databases were systematically searched to identify relevant trials. By investigating these trials, the present review provides updated efficacy and side effect data for promising chemopreventive agents and identifies critical research gaps.

## 2. Methods

A systematic search was conducted in the ClinicalTrials.gov database using the search terms “skin cancer” and “chemoprevention”. A total of 72 studies were identified, which were then filtered by registration date from 1995 to 2022, resulting in 65 studies. Exclusion criteria were then applied to eliminate studies involving other cancer types, resulting in a final analysis of 18 studies that were specific to skin cancer (Table 1).

The PubMed database was also searched using the keywords “skin cancer” and “chemoprevention”, which generated 115 results. Further filtering was performed to select only clinical trials and randomized controlled trials with a registration date from 2010 to 2023, resulting in 55 studies. Exclusion criteria were applied to eliminate studies involving arsenical skin lesions, colorectal, advanced premalignant lesions, lung cancer prevention, subcutaneous pocket infection, surgical site infection, prevention of surgical site infection, gynecological cancer, periocular actinic keratinocytes, breast cancer, second primary tumor, pre-engraftment bloodstream infection, and hepatocellular carcinoma. Studies that did not evaluate drug effects or adverse drug reactions (ADRs), such as those assessing quality of life or predictors of toxicity, were also excluded. The remaining studies were screened, resulting in 21 studies that were considered relevant to skin cancer chemoprevention (Table 2).

## 3. Cancer Types or Conditions of the Clinical Trials

The results of the clinical trial search reveal that most studies focused on NMSC, with a few studies specifically focusing on BCC or SCC. Some studies did not specify cancer types as they utilized precancerous biomarkers or focused on pharmacokinetics or intervention safety. Out of the 18 studies from the ClinicalTrials.gov site, 10 were centered on NMSC prevention, with one each focused on SCC, BCC, AK, and skin immunity, and four were unspecified. No study was focused on melanoma prevention.

The PubMed search results generated more outcomes than ClinicalTrials.gov, and similarly, most studies (10) targeted NMSC, followed by specified BCC (4) and melanoma (3). One study focused on examining how intervention impacted skin immunity. All studies retrieved from PubMed had explicitly stated the targeted conditions.

When the results from both ClinicalTrials.gov and PubMed were combined, NMSC was still the most widely studied cancer type. Fewer trials were conducted for melanoma. Skin immunity was also included as a condition, as it may be a predictor of future skin cancer risk.

## 4. Interventions

Many of the studies found in the ClinicalTrials and PubMed databases were focused on the efficacy of preventive interventions for skin cancers and the adverse effects associated with preventive agents. Most of the studies were conducted with monotherapy, and commonly tested therapeutic agents were nicotinamide, retinoids, non-retinoid topical agents, nonsteroidal anti-inflammatory agents (NSAIDs), and nicotinamide (Table 3). The most frequently studied agent was oral nicotinamide 500 mg, with study durations ranging from 6 to 12 months. Topical 5-fluorouracil 5% strength and topical diclofenac 3% strength were the next most frequently studied agents, with study durations ranging from 2 to 4 weeks and 1 to 9 months, respectively. Celecoxib 200 mg twice daily and aspirin 81 to 100 mg were other agents that appeared multiple times in the search results. Statins, anti-diabetic medications, and dietary supplements were less commonly evaluated but were present in the filtered results (Table 3).

Both topical and systemic (oral) agents were studied. Although a considerable number of studies showed notably positive outcomes, certain studies did not exhibit any significant positive results or were not published. The lack of significant results may suggest the need for a larger sample size, a longer duration of treatment, or a different combination of agents. Overall, further clinical research is required to confirm the effectiveness of these agents in skin cancer prevention and the identification of potential adverse effects.

## 5. Populations

In the search results from the PubMed and ClinicalTrials databases, different target populations were assessed, including healthy individuals and those with an increased risk of skin cancer. The latter included patients with actinic keratoses, a history of NMSC, as well as organ transplant recipients taking immunosuppressants. People at high risk for skin cancer also include patients with the hereditary conditions such as *Xeroderma pigmentosum*. Healthy individuals with sun-damaged skin were included in some trials. Due to the high-impact Veterans Affairs (VA) Keratinocyte Carcinoma Chemoprevention Trials (VAKCCT), elderly individuals with a history of NMSC have received the most attention in identified clinical studies. The higher incidence of skin cancer and the potential to gain benefit from preventive interventions may be reasons that the elderly with a history of NMSC or actinic keratoses have been the most studied populations. Immunosuppressed organ transplant recipients represent a special population that have increased risk for skin cancer, particularly NMSC. It is very common for patients to develop multiple NMSC [24]. Once the first cancer is diagnosed, the patient has a higher risk of developing additional cancer. In contrast, research has paid the least attention to healthy individuals. It is equally important to investigate chemoprevention in healthy individuals for agents with proven safety because the agents can apply to the prevention of future development of skin cancer and to supplement the use of sunscreen in general population.

## 6. Endpoints

A variety of outcomes were measured in the identified trials. The incidence or rate of NMSC was the primary endpoint. Another interesting endpoint that was evaluated in some trials was cost-effectiveness or the ability to save money by using a particular drug. With current research, cost-effectiveness studies may not be the most beneficial due to limited FDA-approved options. To enable early intervention and halt disease progression, crucial endpoints for future research should include biological and immune function markers indicative of future cancer development. Moreover, endpoints such as improved quality of life, symptom relief, and prevention of tumor regression demand further investigation.

## 7. ClinicalTrials.Gov Outcomes

Out of the 18 search results, 9 clinical trials have published their findings (Table 1). For example, topical 5-fluorouracil (5-FU) reduced the risk of SCC for up to one year, but no benefit was observed for BCC (NCT00847912) [25]. Oral celecoxib (200 mg) significantly reduced BCC burden (NCT00023621) [20]. Another trial found that a low dose of eflornithine (or difluoromethylornithine, DFMO) effectively reduced skin biopsy nuclear abnormality in patients with AK (NCT00021294) [26]. The results from some studies retrieved from the ClinicalTrials.gov database were insignificant, such as interventions with high-dose topical tretinoin and oral acitretin on NMSC (Table 1). Some trials have not been completed or did not report data.

## 8. PubMed Database Outcomes

Table 2 lists 20 studies identified from the PubMed database, most of which were focused on agents with anti-inflammatory activities. These agents can be classified into chemotherapy, retinoids, NSAIDs, vitamins, dietary supplements, or agents used for other disorders that can be repurposed for cancer chemoprevention. Their proposed mechanisms of action are shown in Table 3. These agents can be administered orally, topically, or both. The topical delivery provides advantages of higher skin targeting effects and fewer systemic side effects. For studies found in both ClinicalTrials and PubMed, the trials are only listed in Table 1.

## 9. Examples of Chemoprevention Trials

### 9.1. Nicotinamide

Multiple studies evaluated nicotinamide, a form of vitamin B3 or niacin, as a preventative measure for skin cancer. The mechanisms of action for nicotinamide possibly involve increasing DNA repair by blocking UV-induced cellular ATP loss and reducing UV-induced immunosuppression [35,52]. Due to the chemopreventive effects of nicotinamide in preclinical models and earlier small-scale studies in human subjects, a phase III randomized controlled trial was conducted to assess the efficacy of nicotinamide as a chemopreventive agent for NMSC in the Oral Nicotinamide to Reduce Actinic Cancer (ONTRAC) trial, published in 2015 [32]. This trial, with a large sample size of 386 immunocompetent participants, was able to conclude statistically significant differences between nicotinamide use and placebo for decreasing rates of new-onset AK and NMSC [32]. Based on the evidence, nicotinamide is recommended by up to 76.9% of Mohs surgeons for NMSC prevention [53].

Later, at least two clinical trials evaluated the effects of oral nicotinamide in immunocompromised individuals. One phase II randomized controlled trial of nicotinamide was conducted to evaluate the skin cancer chemoprevention in renal transplant recipients [33]. This study could not conclude statistically significant results due to a small sample size (*n* = 22), although they reported reductions in AKs in the nicotinamide group compared to the placebo group [33]. More recently, given nicotinamide’s potential activity against immunosuppression, the Oral Nicotinamide to Reduce Actinic Cancer after Transplant (ONTRANS) trial was conducted on solid-organ transplant recipients with a history of multiple NMSC who received nicotinamide for 12 months [34]. The incidence of NMSC was nearly identical in nicotinamide and placebo groups. Therefore, the use of nicotinamide as a preventative measure for NMSC produced negative results in immunosuppressed solid-organ transplant recipients [32]. Although most studies supported the safety of nicotinamide as a chemopreventive agent with little to no side effects at doses as high as three grams daily [32], in the ONTRAC trial, patients who received nicotinamide, compared to the patients who received placebo, had significantly more mucocutaneous infections (lip, mucosal, nail, skin, and wound infections, as well as paronychia and sinusitis) [54].

The potential efficacy of nicotinamide was also shown in individuals living in arsenic-contaminated areas based on preclinical studies [55].

Nicotinamide is a substrate of nicotinamide N-methyltransferase (NNMT), which catalyzes the N-methylation of nicotinamide and regulates its level. In the past two decades, NNMT has been shown involved in carcinogenesis and tumor progression, including skin cancer [56]. The interaction of NNMT and nicotinamide needs further investigations.

### 9.2. NSAIDs

Cyclooxygenase-2 (COX-2) and its metabolic product prostaglandin E_2_ (PGE_2_) can be induced by UV radiation and play important roles in skin inflammation and carcinogenesis [57]. Nonsteroidal anti-inflammatory drugs (NSAIDs) exhibit anti-inflammatory effects by inhibiting COX-2 selectively or non-selectively and inhibiting the production of PGE_2_ and have been investigated in multiple trials. NSAIDs include celecoxib, diclofenac, etodolac, rofecoxib, ibuprofen, naproxen, indomethacin, MF-tricyclic, sulindac, piroxicam, and aspirin. Since the anti-inflammatory activity of NSAIDs is believed to be mediated by the inhibition of COX-2, while gastrointestinal toxicity is due to COX-1 inhibition, several COX-2 selective inhibitors, such as celecoxib, were developed to avoid gastrointestinal adverse reactions [58]. None of the clinical studies could confirm that NSAIDs have significant effects as skin cancer chemopreventive agents, but there were other findings to note. One study focused on NSAIDs’ effects on BCC chemoprevention in mice and humans that are carriers of the mutant PTCH1, a receptor of the hedgehog pathway [20]. Oral celecoxib, a selective COX-2 inhibitor, was given to the human participants at a dose of 200 mg twice daily. Although celecoxib showed a 75% decrease in BCC tumor burden in mice, in the human trial, the effects of oral celecoxib in reducing BCC burden in all subjects were insignificant. However, when considering only 60% of the patients with less severe diseases (<15 BCCs at study entry), celecoxib significantly reduced the BCC numbers and burden. In another study, research on celecoxib for the chemoprevention of NMSC was conducted [36]. Participants were randomized and began treatment with either celecoxib or placebo and then evaluated at 3, 6, 9, and 11 months after randomization. No difference in AKs was found at nine months of treatment, but at 11 months, there was a significant reduction in the mean NMSCs per patient in the celecoxib group. After adjusting for age, sex, skin cancer history, etc., the results were still found to be statistically significant. However, the results were inconclusive due to the early termination of the study by the FDA based on another finding of an association between a COX-2 inhibitor and an increased risk of cardiovascular adverse events [36]. The efficacy results are consistent with previous observational studies, however, due to potential increased cardiovascular risks, celecoxib’s use as a chemopreventive agent could be limited.

A prospective cohort study examined the association between NSAIDs and NMSC in veterans with a higher risk of skin cancer [38]. The investigators hypothesized that NSAIDs and COX-2-selective inhibitors would provide transient protection against keratinocyte carcinoma, with COX-2-selective inhibitors having greater effects. The study participants were all from the Veterans Affairs Topical Tretinoin Chemoprevention Trial (VATTC), with 1131 veterans recruited. During a median follow-up time of 2 years for BCC and 2.5 years for SCC, 472 occurrences of BCC and 309 occurrences of SCC were observed. Time-fixed analyses and time-varying analyses were performed to avoid potential confounding bias. The time-fixed analyses produced a negative association but were determined not to be valid, and the time-varying analyses produced null results. Overall, this study did not prove a negative association between the use of NSAIDs and the risk of NMSC. The study concluded that the inverse dose response observed in the current study and in prior studies may be an artifact of analytic method.

Since regular use of the non-selective COX-1/COX-2 inhibitor aspirin (acetylsalicylic acid) has been associated with the risk reduction of multiple cancer types [57], the chemopreventive properties of aspirin were studied for skin cancer. One study tested the effect of aspirin on melanoma in elderly patients, but the results did not provide strong evidence that aspirin was associated with a reduced incidence of melanoma [37]. Another study examined the effects of aspirin alone or combined with folic acid in a randomized, double-blind, placebo-controlled clinical trial [39]. A total of 1121 patients were enrolled in the trial of prevention of colorectal adenomas, which was repurposed for BCC. BCC was confirmed by a blinded review of the pathology reports. Although aspirin and folic acid failed to show statistically significant effects on reducing the risk of BCC, subgroup analysis indicates that BCC risk was lower with aspirin use in those with previous skin cancer, while folic acid was unrelated to BCC incidence. Consistently, a retrospective study (2010–2018) conducted using the Humana Health Insurance database concluded that aspirin use was associated with a significantly decreased risk of BCC [59]. Therefore, given the high incidence and cost of BCC treatment, the low cost of aspirin and its widely accepted use may promote its preventive use for this type or other types of skin cancer.

Given the systemic adverse effects observed for most NSAIDs, topical delivery has been investigated for achieving a local preventive activity. Diclofenac sodium 3%, in combination with hyaluronic acid 2.5% (diclofenac 3%/HA 2.5%; Solaraze^®^, Fougera Pharms) is the only NSAID approved in the United States for the topical treatment of AK lesions (for review, see [60]). The topical Solaraze^®^ proved to be effective for patients with existing AK. It is well tolerated, with skin irritation as the main side effect [57]. Another topical COX-2 inhibitor is piroxicam, which is a nonspecific COX-1 and COX-2 inhibitor, with higher inhibitory activity (10-fold) for COX-1. Finally, 1% piroxicam gel, topically applied daily for 12 weeks, was shown to effectively induce the complete regression of 48% of evaluated AKs, with an adverse effect of only skin irritation [40]. Thus, the topical application of NSAIDs is promising for providing cancer preventive efficacy with minor side effects.

### 9.3. Retinoids

Since the retinoid signaling pathway plays an important role in organ homeostasis and carcinogenesis, the natural and synthetic vitamin A derivatives, retinoids, may be effective for the prevention and the treatment of several types of cancer, including skin cancer (for review, see [61]). Initially, oral retinoids demonstrated efficacy as chemopreventive agents against NMSC and other types of cancer [24,48]. Therefore, there was a trend during the 1960s and 1970s to develop synthetic retinoids for cancer prevention and treatment [62]. Oral isotretinoin, acitretin, and etretinate have been reported to reduce BCCs in patients with *Xeroderma pigmentosum*, organ transplantation recipients, and individuals with basal cell nevus (Gorlin) syndrome (BCNS) [48]. However, the protective effects were lost after the therapy was discontinued. Lower doses with fewer side effects were ineffective [24]. Long-term and high-dose use of systemic retinoids has been associated with significant dose-dependent side effects [62]. For example, published in 2012, systematic use of acitretin for 2 years in nontransplantation patients at high risk for NMSC did not show statistically significant reduction in the rate of new NMSC, while the patients who received acitretin reported significantly more mucositis and skin toxicities compared to the placebo group [21]. Therefore, systematic use of retinoids is limited in the general population with no or few skin cancers.

Topical use of retinoids, e.g., tretinoin, has been used for decades for the treatment of acne and photoaging, without systematic side effects [24]. Thus, topical retinoids have been investigated in the Veterans Affairs Topical Tretinoin Chemoprevention (VATTC) Trial [24]. In this trial, 1131 patients were given topical 0.1% tretinoin or a matching vehicle control for 1.5–5.5 years. Reported in 2012, the primary outcomes, the rates of new BCC and SCC, did not differ significantly for the treatment. The tretinoin group showed worse cutaneous symptoms. This trial concluded that in high-risk patients, high-dose topical tretinoin was ineffective at reducing risk of NMSC.

Tazarotene (Tazorac^®^, Allergan, Irvine, CA) is a topical retinoid with relative specificity for retinoic acid receptor (RAR)-β and RAR-γ receptors. A randomized, double-blind, vehicle-controlled study in patients with basal cell naevus syndrome (BCNS) evaluated the efficacy of topically applied tazarotene for BCC chemoprevention (*n* = 34 subjects), along with an open-label trial evaluating tazarotene’s efficacy for chemotherapy of BCC lesions (*n* = 36 subjects) for a maximum follow-up period of 3 years. Only 6% of patients had a chemopreventive response, and only 6% of treated BCC target lesions were clinically cured. Thus, this study provides no evidence for either chemopreventive or chemotherapeutic effect of tazarotene against BCCs in patients with BCNS. Therefore, despite the robust effects of topical retinoids in preclinical studies, they failed to demonstrate the effects in clinical studies.

### 9.4. 5-Fluorouracil (5-FU)

5-FU is a chemotherapy agent that belongs to a class of antimetabolites. It is used topically to treat skin cancer and AK. An earlier study that is frequently referenced in prevalent skin cancer publications is Predictors of squamous cell carcinoma in high-risk patients in the VATTC trial [41]. The study followed participants, mostly men with a median age of 72 with a history of heavily sun damaged skin. The subjects were required to have at least two forms of NMSC in the five years prior to their enrollment and had a follow up period of approximately four years. The purpose of this study was to find alternative preventive measures other than systemic retinoids, which have significant toxicity. A total of 1131 participants were screened, and of those participants, 23% developed at least one new SCC. The most important predictors of new SCC were identified as the number of prior carcinomas, the number of prior in situ carcinomas, the number of AKs prior to the study, the amount of sun exposure, and a history of 5-FU use. Participants that fell in the category of having many predictors had a significantly higher hazard ratio than those in the category of least predictors. Through a univariable analysis, all predictors were found to be statistically significant, while total sun exposure was found to have a greater association with newly developed SCC. The study concluded that a history of 5-FU use was strongly associated with an increased risk of future SCCs. On the other hand, using angiotensin-converting enzyme inhibitors or angiotensin receptor blockers reduced risk of SCC development. Furthermore, the study concluded that the administration of high-dose topical tretinoin was ineffective in diminishing the risk of NMSC.

Topical 5-FU (5%) has been investigated in multiple clinical trials of skin cancer chemoprevention, and most produced positive results. In the randomized Veterans Affairs Keratinocyte Carcinoma Chemoprevention (VAKCC) Trial of 932 veterans at high risk for NMSC, a 2–4-week duration of topical 5-FU reduced the risk of SCC for 1 year, but no effects were seen on BCC incidence in the first year [25]. There were no effects on SCC or BCC incidence at 4 years. Due to a potent chemopreventive effect in immunocompetent patients, a recent phase II open-label randomized controlled trial compared topical 5-FU, 5% imiquimod, and sunscreen in organ transplant recipients [45]. The pilot feasibility study suggested that topical 5-FU may be superior to imiquimod and sunscreen in AK clearance and prevention. Thus, 5-FU topical chemoprevention should be further investigated in SCC/AK prevention for immunocompromised patients.

The oral prodrug of 5-FU, capecitabine, was examined for the prevention and treatment of AK and NMSC. A systemic review indicated that capecitabine treatment may be associated with a decrease in the incidence of SCCs in organ transplant recipients [63]. ADRs, including fatigue, nausea, vomiting, diarrhea, elevated creatinine level, hand–foot syndrome, hyperuricemia, weight loss, anemia, and cardiomyopathy, limited the duration of chemoprevention in several patients.

### 9.5. Difluoromethylornithine (DFMO)

Difluoromethylornithine (DFMO), an inhibitor of ornithine decarboxylase, inhibits polyamine synthesis, which can be increased in UV-induced skin cancer [64]. A number of clinical trials have evaluated the effects of systemic DFMO, alone or in combination with other agents, for preventing skin cancer. Although the benefit of DFMO is promising, it has been reported to cause some side effects, such as hearing loss [64]. Therefore, topical application of DFMO is an option for reduced systemic effects. A previous study (NCT00021294) investigated the efficacy of topical administration of DFMO (10%), triamcinolone (1%), and the combination of DFMO plus triamcinolone for the reduction of cell nuclear abnormality in moderately sun-damaged skin [26]. Eucerin^®^ (Beiersdorf Inc., Hamburg, Germany), a commercially available cream, was used as a vehicle. A total of 102 participants with sun-damaged skin on their posterolateral forearms were recruited for this study, and 185 skin biopsies were collected, with 16,395 nuclei recorded. High-resolution imagery of nuclei was utilized to assess the reduction of nuclei post-treatment and to compare them to baseline levels. Four treatment groups were established, including applying DFMO + Eucerin^®^, DFMO + triamcinolone, triamcinolone + Eucerin^®^, and Eucerin^®^ + Eucerin^®^ as a placebo. Participants applied 1 inch of cream to their forearms once daily throughout the study. The study found that applying these treatments resulted in a significant reduction in cell nuclear abnormality by 15–20%. These findings suggest that low-dose topical applications of DFMO, triamcinolone, and the combination of DFMO plus triamcinolone may effectively improve nuclear abnormality in moderately sun-damaged skin.

Since topical DFMO and topical diclofenac (an NSAID) as monotherapy have demonstrated chemopreventive activity against SCC, a phase IIB randomized trial of topical DFMO and diclofenac was conducted to evaluate the effects on sun-damaged skin in 136 patients who completed the study over three months. The goal was to examine whether the combination was more effective than monotherapy in reversing karyometric average nuclear abnormality, a predictor for SCC. However, the nuclear abnormalities increased in all three groups. The addition of topical DFMO to topical diclofenac did not enhance its activity but induced cutaneous inflammation as a side effect [27]. Based on this study, questions were raised regarding the efficacy of these agents, the status of cancer risk in the study population, and the validity of nuclear abnormalities as a marker [65].

### 9.6. Polyunsaturated Fatty Acids (PUFAs)

A study investigated the potential protective effect of the dietary supplement omega-3 polyunsaturated fatty acids (PUFAs) against photo-immunosuppression caused by solar UV radiation [30]. A total of 79 healthy female participants between the ages of 18 and 60 years were randomly assigned to either receive an oral placebo control lipid supplement or oral omega-3 PUFA (70% EPA and 10% DHA). The participants received nickel contact hypersensitivity patches to assess changes in photo-immunosuppression. After supplementation with either the control or PUFA, nickel was applied to the participants’ skin sites pre-exposed to three consecutive days of solar-simulated radiation (SSR) at a dose of 3.8 J/cm^3^ and three unexposed control sites. The study found that omega-3 PUFAs were protective against photo-immunosuppression by lowering 50% of immunosuppression, measured by nickel contact hypersensitivity. The significance of this study lies in providing a potential solution to minimize skin damage caused by sunlight that can ultimately lead to skin cancer. This is particularly important because conventional sunscreens are inadequate in protecting against photo-immunosuppression compared to UV-induced erythema and are often misused or underutilized. Future well-designed human studies are needed to evaluate the effects of PUFAs.

### 9.7. Antidiabetic Drugs

Patients with type II diabetes show a higher risk of NMSC. These patients may benefit from the use of the commonly prescribed antidiabetic drug metformin to reduce the risk of NMSC and other types of cancer [47]. A secondary analysis of patients enrolled in the VAKCC trial was conducted to compare the risk for NMSC development between metformin users and non-users (NCT00847912). Metformin-users had a significantly lower risk for SCC and BCC compared to non-users [66]. Another antidiabetic drug pioglitazone showed robust efficacy in preventing SCC in preclinical models [51], but no clinical trial data have been published yet. Future clinical trials are needed to answer the question of whether antidiabetic drugs are effective for skin cancer chemoprevention.

## 10. Safety Issues

Since the target population for cancer chemoprevention is healthy individuals with increased cancer risk, pharmacological agents that can be used for preventive purposes should be both effective and safe. Although the primary endpoints are usually efficacy, many clinical studies have reported side effects besides chemopreventive efficacy. Although some agents have demonstrated statistically significant efficacy, safety issues emerged after long-term and/or high-dose administration of these agents.

Among the chemopreventive interventions tested, trials using COX-2 inhibitors seemed to have the greatest risk to patients. Nearly all articles reported general adverse effects of these medications as well as serious cardiovascular effects. Common side effects associated with COX-2 inhibitors include infections, gastrointestinal disorders, musculoskeletal effects, and skin disorders. Cardiovascular effects included hypertension, myocardial infarction, stroke, congestive heart failure, or cardiovascular deaths. To better evaluate the safety profile, one trial performed a safety analysis that incorporated all randomly assigned patients that took at least one dose of the trial medication, placebo, or celecoxib [36]. The adverse events were compared by using either a chi-squared or Fisher exact test. Of the 183 subjects (87 in the celecoxib group and 96 in the placebo group), a total of 16 subjects reported experiencing severe adverse events, including 9 from the celecoxib group and 7 from the placebo group, with no deaths reported among all the subjects. The analysis concluded no significant differences between the placebo and the experimental groups.

Numerous studies have demonstrated toxicity associated with retinoids, particularly with systemic use [62]. Long-term retinoid therapy, especially at high doses, has been associated with teratogenicity [67], skeletal toxicity, such as the calcification of tendons and ligaments around joints, and hyperostosis of the spine, as well as osteoporosis [62]. Topical forms of retinoids can also induce toxicity, such as skin irritation. The safety issues may be related to a trend of less recent clinical trials on retinoids.

On the other hand, vitamins or dietary supplements, e.g., nicotinamide, are safe for long term use. Future studies should aim for proving the efficacy of these natural products.

## 11. Limitation of the Current Review

The limitations of this review include a narrow range of database searches using only two keywords. Additionally, this review could have included a more in-depth analysis of the studies’ methodologies to assess the quality of evidence. This review does not include intensive mechanisms of action or molecular targets for chemopreventive agents. Furthermore, this review’s focus on English-language studies may have resulted in the exclusion of relevant studies published in other languages.

## 12. Conclusions

This review provides an overview of the current state of the clinical research on the chemoprevention of skin cancer. While a significant proportion of the studies included in this review yielded positive efficacy results with statistical significance, the findings are not consistent when similar studies were conducted in different populations. For example, the results in the clinical trials for nicotinamide were not totally reproducible, and the data so far cannot provide sufficient evidence for drug efficacy due to doubts in the data analyses [68]. Further research is required to confirm the effectiveness of these agents in skin cancer prevention in various patient populations and to identify the complete profiles of adverse effects. The available evidence from multiple trials, including those found in the PubMed and ClinicalTrials databases, indicate that skin cancer chemoprevention is a largely under-researched area. Despite the high prevalence of skin cancer and the limited treatment options available, there has been a lack of attention given to this critical area of research, since cancer chemoprevention trials require relatively longer follow-up time compared to cancer treatment trials. Although certain skin cancer conditions, such as BCC, have been evaluated in several studies, others, including melanoma, have not received adequate attention. There is a significant need for future research studies to address the need for skin cancer chemoprevention particularly in subgroups of patients who are immunocompromised. Since systemic use of chemopreventive agents, such as COX-2 inhibitors and retinoids, can cause side effects, topical drug delivery shows advantages and should receive more attention. Systemic use of vitamins and dietary supplement should be a safer option. To address the safety issues of cancer chemopreventive agents, the effort should be directed to identify chemopreventive dietary supplements and to repurpose FDA-approved agents.

## Figures and Tables

**Table 1:** Search results from the ClinicalTrials.gov database.

ID	Condition	Intervention	Subject	Endpoint	Size	Results
NCT00023621	BCC	Oral celecoxib	Patients with history of BCC	Rate of BCC	60	Celecoxib significantly reduced BCC number and burden [20].
NCT00644384	NMSC	Oral acitretin	With history of NMSC	Rate of new NMSC; surrogate biomarkers	130	No results published
NCT00003611	NMSC	Oral acitretin	With history of skin cancers with organ transplantation	Rate of new NMSC; surrogate biomarkers	70	Acitretin showed benefit but not significant; the patients who received acitretin reported significantly more mucositis and skin toxicities compared to the patients who received placebo [21].
NCT00007631	NMSC	Topical tretinoin	With history of NMSC	Rate of NMSC	1131	High-dose topical tretinoin was ineffective at reducing risk of NMSC [22,23,24].
NCT00847912	NMSC	5-FU topical	Veterans with history of NMSC	Rate of NMSC	954	Risk of SCC reduction was seen in the first year only; risk of BCC reduction in the first year was not significant [25].
NCT00021294	NMSC	Topical DFMO combined with triamcinolone	Patients with AK	Rate of NMSC	102	The low-dose topical drug interventions were effective in reducing skin biopsy nuclear abnormality [26].
NCT00601640	Other	DFMO combined with diclofenac	Individuals with skin sun damage	Nuclear marker	156	The addition of topical DFMO to topical diclofenac did not enhance its activity [27].
NCT00204789	Other	Oral DFMO	Organ transplant recipients	Safety; targets of DFMO	52	No significant effect for DFMO [28]; oral DFMO at 500 mg/m^2^/day was safe and tolerable and resulted in significant inhibition of phorbol ester-induced skin ODC activity [29].
NCT01032343	Skin immunity	Omega-3 polyunsaturated fatty acids (PUFA)	Healthy volunteers	Nickel contact hypersensitivity	79	Oral PUFAs abrogated photoimmunosuppression in human skin, providing additional support for their chemopreventive role [30].
NCT01447355	Other	Oral cholecalciferol (vitamin D)	Healthy subjects with insufficient serum levels of 25-hydroxyvitamine D	Changes in vitamin D receptor expression; skin differentiation biomarkers; safety and tolerability	25	High-dose cholecalciferol supplementation raised serum VD metabolite levels and CYP24 mRNA and caspase-14 levels in the skin [31].
NCT00002811	AK	Liposomal T4N5 lotion		Rate of AK	30	No results published
NCT00089180	NMSC	Liposomal T4N5 lotion	Renal transplant recipients with history of NMSC	Rate of NMSC	100	No results published
NCT03769285	NMSC	Oral nicotinamide	Solid organ transplant recipients	Rate of NMSC	120	Ongoing
NCT04091022	NMSC	Topical diclofenac and topical DFMO	With history of NMSC	Rate of NMSC	138	No results published
NCT02636569	NMSC	Topical diclofenac	History of NMSC	Biomarkers in skin biopsies	24	No results published
NCT03210740	NMSC	AM001 Cream (topical Potassium dobesilate)	Patients with AK	Clearance of AK	30	No results published
NCT02347813	SCC	Oral pioglitazone	Patients with history of frequent occurrence of SCC	Rate of SCC	12	No results published
NCT05159752 NCT05370235	Other	Afamelanotide	XP patients	Safety and efficacy on skin damage	6	Recruiting

NMSC: non-melanoma skin cancer; BCC: basal cell carcinoma; SCC: squamous cell carcinoma; AK: actinic keratosis; XP: *Xeroderma pigmentosum*; DFMO: difluoromethylornithine; PUFA: polyunsaturated fatty acid; 5-FU: 5-fluorouracil.

**Table 2:** PubMed database outcomes.

PMID	Condition	Intervention	Subject	Endpoint	Size	Results
26488693	NMSC	Oral nicotinamide	With history of NMSC	New NMSC	386	Oral nicotinamide was safe and effective in reducing the rates of new NMSC and actinic keratoses in high-risk patients [32].
27061568	NMSC	Oral nicotinamide	Organ transplant recipients with history of NMSCs	Rate of NMSC	22	Oral nicotinamide was associated with a statistically nonsignificant relative difference in the rate of NMSCs and statistically nonsignificant reduction in AK [33].
36856616	NMSC	Oral nicotinamide	Organ transplant recipients with history of NMSCs	Rate of NMSC	158	Oral nicotinamide did not lead to lower numbers of NMSC or AK in immunosuppressed solid-organ transplant recipients [34].
30244097	Skin immunity	Oral nicotinamide	Immunocompetent patients who had NMSCs	Immunological markers	78	The study found significant decrease in the number of macrophages in keratinocytes that arose in patients receiving nicotinamide compared to placebo [35].
20051370	BCC	Oral celecoxib	PTCH1(+/−) patients with BCNS	Rate of BCC	60	Celecoxib decreased the development of new BCCs in all subjects, but it did not reach statistical significance [20].
21115882	NMSC	Oral celecoxib	History of AK	Incidence of SCC, BCC, and AK	240	Celecoxib might be effective for prevention of SCCs and BCCs in individuals who had extensive actinic damage and were at high risk for development of NMSC [36].
35395069	Melanoma	Oral aspirin	Elderly	Rate of melanoma	19,114	Aspirin was not associated with a reduced risk of invasive melanoma in older individuals [37].
21688346	NMSC	NSAIDs	Veterans with higher risk of NMSC	Rate of SCC and BCC	728	This study did not identify a negative association between NSAIDs and keratinocyte carcinomas [38].
29570772	BCC	Aspirin and/or folic acid orally	Diagnosed with colorectal adenomas	Rate of BCC	1121	Neither aspirin nor folic acid treatment had a statistically significant effect on risk of BCC. Subgroup analysis suggested that chemopreventive NSAIDs may be specific to those at high risk for BCC [39].
20000874	NMSC	Topical piroxicam	With history of AK	Actinic Keratosis Erythema Scale Atrophy score	31	The use of piroxicam 1% gel for 90 days induced complete regression in 48% of evaluated actinic keratoses [40].
23348836	SCC	5-FU; angiotensin-converting enzyme inhibitors or angiotensin receptor blockers	Veterans with history of NMSC	Rate of SCC	1131	Key risk factors for additional SCCs in patients with multiple prior NMSC was identified [41].
29505863	NMSC	Topical 5-FU	Veterans with high risk of NMSC	Cost	932	There was a significant cost savings for patients treated with 5-FU [42].
33795573	NMSC	Topical 5-FU	History of AK	Rate of NMSC	932	A single 2- to 4-week course of topical 5-FU to the face and ears decreased overall biopsy rates for 1 year. SCC biopsy yield was decreased in the first year after treatment. There was a nonsignificant trend toward increased BCC biopsy yield [43].
30896781	BCC	Topical 5-FU	Veterans with high risk of NMSC	Rate of BCC	932	5-FU might be effective for the prevention of superficial subtype of BCCs even though there was no effect on BCCs overall [44].
34988975	SCC	Topical 5-FU	Organ transplant recipients	Rate of	40	Trials of topical AK treatments for SCC chemoprevention are feasible [45].
21463984	NMSC	Topical potassium dobesilate	History of AK	Lesions of actinic keratosis	30	The use of potassium dobesilate 5% cream for 16 weeks induced complete regression in 70% of evaluated actinic keratoses [46].
35533029	NMSC	Metformin and sulfonylureas	Diabetic patients with history of NMSC	Rate of NMSC	932	Diabetic patients at high risk for KC might benefit from the use of metformin versus sulfonylureas [47].
24441673	BCC	Topical tazarotene (a retinoid)	Patients who have BCNS	Rate of BCC	34	The study provided no evidence for either chemopreventive or chemotherapeutic effect of tazarotene against BCCs in patients with BCNS [48].
24614012	Melanoma	Oral lovastatin for 6 months	Subjects with at least two clinically atypical nevi	Biomarkers of melanoma	80	There were no effects. Further research into pathogenesis of melanoma and other chemopreventitive agent is needed [49].
29691233	Melanoma	Sulforaphane with administration of oral broccoli sprout extract	Patients had at least 2 atypical nevi and a prior history of melanoma	Safety; plasma and skin drug levels; biomarkers	17	Oral BSE-SFN was well-tolerated at daily doses up to 200 µmol and achieved dose-dependent levels in plasma and skin. Efficacy studies may be performed in the future [50].
20103724	Unspecified	Topical perillyl alcohol (POH)	Individuals with sun-damaged skin.	Skin histopathologic scores and nuclear chromatin pattern	89	Karyometric analyses could detect a modest effect of POH in sun-damaged skin. Improved delivery into the epidermis may be necessary [18].

**Table 3:** Summary of chemopreventive agents identified from the ClinicalTrials and PubMed databases.

Drug Class	Mechanism of Action	Drug	Route of Administration	Dose/Strength and Duration
Chemotherapy	Antimetabolite	5-Fluorouracil	Topical	5%; 4 weeks [42]
				5%; 2–4 weeks [44]
				5%; 4 weeks [43]
Retinoids	PAR inhibition	Acitretin	Oral	25 mg; 2 years [21]
Retinoids	PAR inhibition	Tretinoin	Topical	0.1%; 1.5–5.5 years [24]
Retinoids	PAR inhibition	Tazarotene	Topical	0.1%; 3 years [48]
NSAID	Anti-inflammation	Celecoxib	Oral	200 mg; 2 years [20]
				200 mg; 9 months [36]
NSAID	Anti-inflammation	Piroxicam	Topical	1%; 12 weeks [40]
NSAID	Anti-inflammation	Diclofenac	Topical	3%; 6 months [27]
NSAID	Anti-inflammation	Aspirin	Oral	81 or 325 mg; 3 years [39]
				100 mg; 5–7 years [37]
DFMO	Ornithine decarboxylase (ODC) inhibitor	Eflornithine	Topical	10%; 6 months [27]
				10%; 6 months [26]
Vitamin B3	DNA repair/inhibiting immunosuppression	Nicotinamide	Oral	500 mg; 12 months [32]
				500 mg; 6 months [33]
				500 mg; 12 months [35]
Vitamin D	Skin cell differentiation	Cholecalciferol	Oral	50,000 IU; 8–9 weeks [31]
Bacteriophage T4 endonuclease 5	DNA repair	T4N5	Topical	12-month; no results
Statin	Anti-inflammation	Lovastatin	Oral	40–80 mg; 6 months [49]
Anti-diabetic medication	PPARγ agonist	Pioglitazone	Oral	15–30 mg; 5½ months [51]
Anti-diabetic medication	Multiple	Metformin	Oral	2.8 years [47]
Anti-diabetic medication	Unknown	Sulfonylurea	Oral	2.8 years [47]
Limonene derivative	Unknown	Perillyl alcohol	Topical	0.3% or 0.76%; 12 weeks [18]
Psoriasis medication	FGF inhibitor	Potassium dobesilate	Topical	5%; 16 weeks [46]
Dietary supplement	Anti-inflammation	Omega-3	Oral	5 mg; 3 months [30]
Dietary supplement	Anti-inflammation	Sulforaphane	Oral	50, 100, or 200 μmol; 28 days [50]

PAR: retinoic acid receptors.
